# Persistent fifth aortic arch: a comprehensive literature review

**DOI:** 10.3389/fped.2023.1183345

**Published:** 2023-06-26

**Authors:** Haiyan Shan, Xiaolan Du, Guangrong Zheng, Tengfei Ke, Chengde Liao, Haiyan Yang

**Affiliations:** ^1^ Department of Radiology, Yan'an Hospital of Kunming City, Yan'an Hospital Affiliated to Kunming Medical University, Kunming, China; ^2^ Department of Radiology, Yunnan Cancer Hospital, The Third Affiliated Hospital of Kunming Medical University, Kunming, China; ^3^ Department of Ultrasound, Chongqing General Hospital, Chongqing, China

**Keywords:** persistent fifth aortic arch, classification, diagnosis, imaging, treatment

## Abstract

Persistent fifth aortic arch (PFAA) is an extremely rare congenital cardiovascular anomaly resulting from the failure of the fifth aortic arch to degenerate during embryonic development; it is often associated with various other cardiovascular anomalies. Despite being first reported by Van Praagh in 1969, there have been only a few individual case reports. Owing to its rarity and lack of comprehensive understanding, PFAA is often misdiagnosed or missed diagnosed during clinical. Thus, this review aimed to summarise the embryonic development, pathological classification, imaging diagnosis, and clinical treatment of PFAA to improve its overall understanding, ultimately helping in accurate diagnosis and treatment.

## 1. Introduction

Persistent fifth aortic arch (PFAA) is widely recognised as a rare congenital cardiovascular malformation that results from the failure of the fifth aortic arch to degenerate during embryonic development (1). It was first reported by Van Praagh through autopsy in 1969 (2). PFAA has an extremely low incidence rate, with Gerlis (3) reporting an incidence of only 0.3%. Clinical symptoms of PFAA are nonspecific and mainly depend on the combination of other deformities and their haemodynamic changes. PFAA is usually confirmed during the neonatal or infant period, with only a few cases reported in adults (4). PFAA is earliest classified by the Weinberg classification (5). Subsequently, some new classifications have been reported according to the proximal and distal connections of the vessel and the direction of blood flow (6, 7). Due to the rarity of PFAA and the fact that only individual cases are reported, misdiagnosis and missed diagnosis are common in clinical practice (8–10). Thus, this review aimed to improve the comprehensive understanding of PFAA by summarising its embryonic development, pathological classification, imaging diagnosis, and clinical treatment to improve diagnostic accuracy and treatment.

## 2. Mechanisms of embryonic development

**Figure 1:**
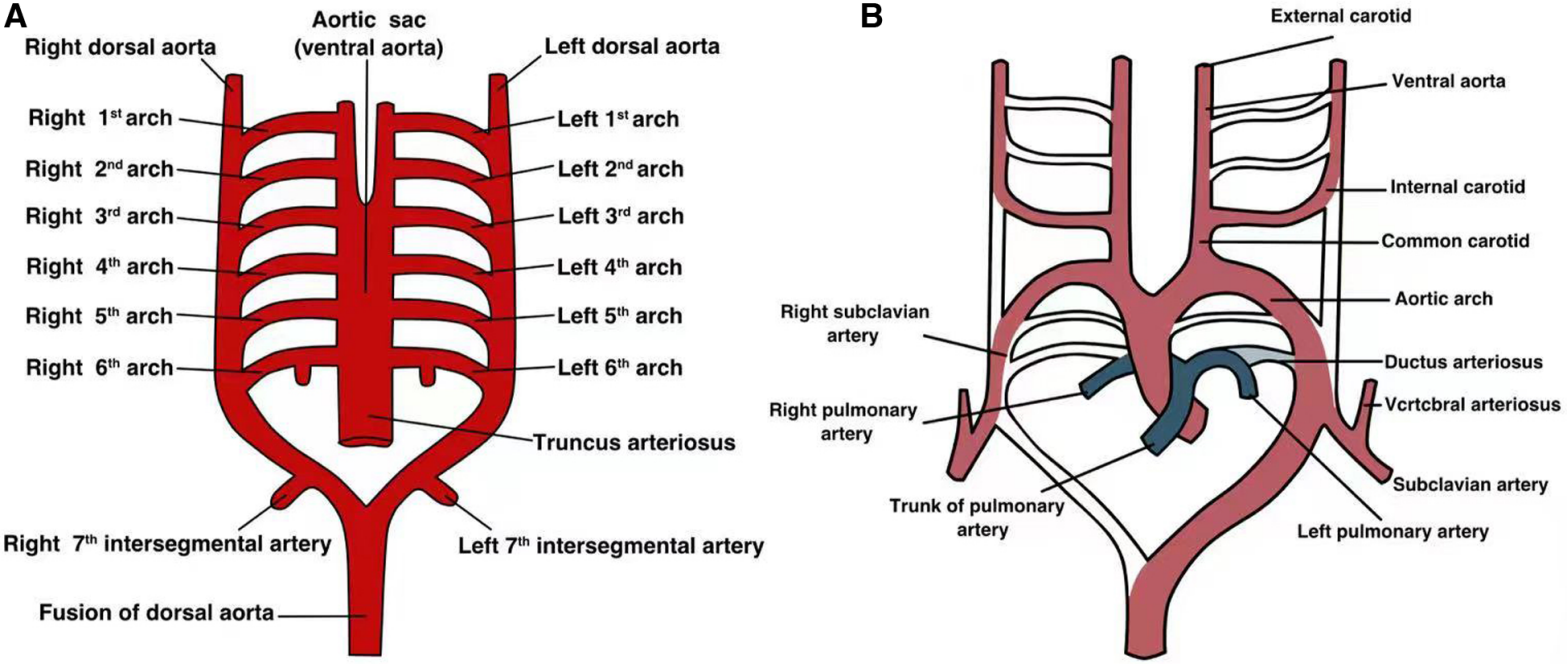
The developmental process of each pair of aortic arches in the embryonic stage. (**A**) Each pair of arterial arches connects to the ventral aorta and dorsal aorta; (**B**) a simple graph of the development of each pair of arterial arches in the embryo.

The aortic arch develops from the aortic sac. During normal embryonic development, six pairs of branchial arches originate from the pharyngeal arch and extend to the dorsal aorta (Figure 1). However, these arches do not always persist and undergo a sequence of orderly digestion and absorption processes between embryonic weeks 3–8 (11). The embryonic development of the arch is asymmetrical. Typically, the first and second branchial arches evolve into the transcranial artery (maxillary and hyoid arteries), whereas the third branchial arch exists permanently, forming the left and right common carotid arteries and part of the internal carotid artery, with their roots connected to the aortic sac. The right fourth branchial arch connects to the right seventh intersegmental artery and then to the right third branchial arch, ultimately forming the brachiocephalic trunk. The left fourth branchial arch connects to the distal and proximal left dorsal aortas to form the horizontal aorta. The right sixth branchial arch forms the right pulmonary artery, whereas the left sixth branchial arch forms the ducts or arterial ductal ligament.

During normal embryonic development of the arterial system, the bilateral fifth branchial arch is immediately absorbed after its development. However, PFAA develops if any unilateral and/or bilateral branchial arches fail to degenerate or completely degenerates and participates in vascular ring formation (12), resulting in hypoplastic blood vessels (13, 14).

## 3. Clinical classification

**Figure 2:**
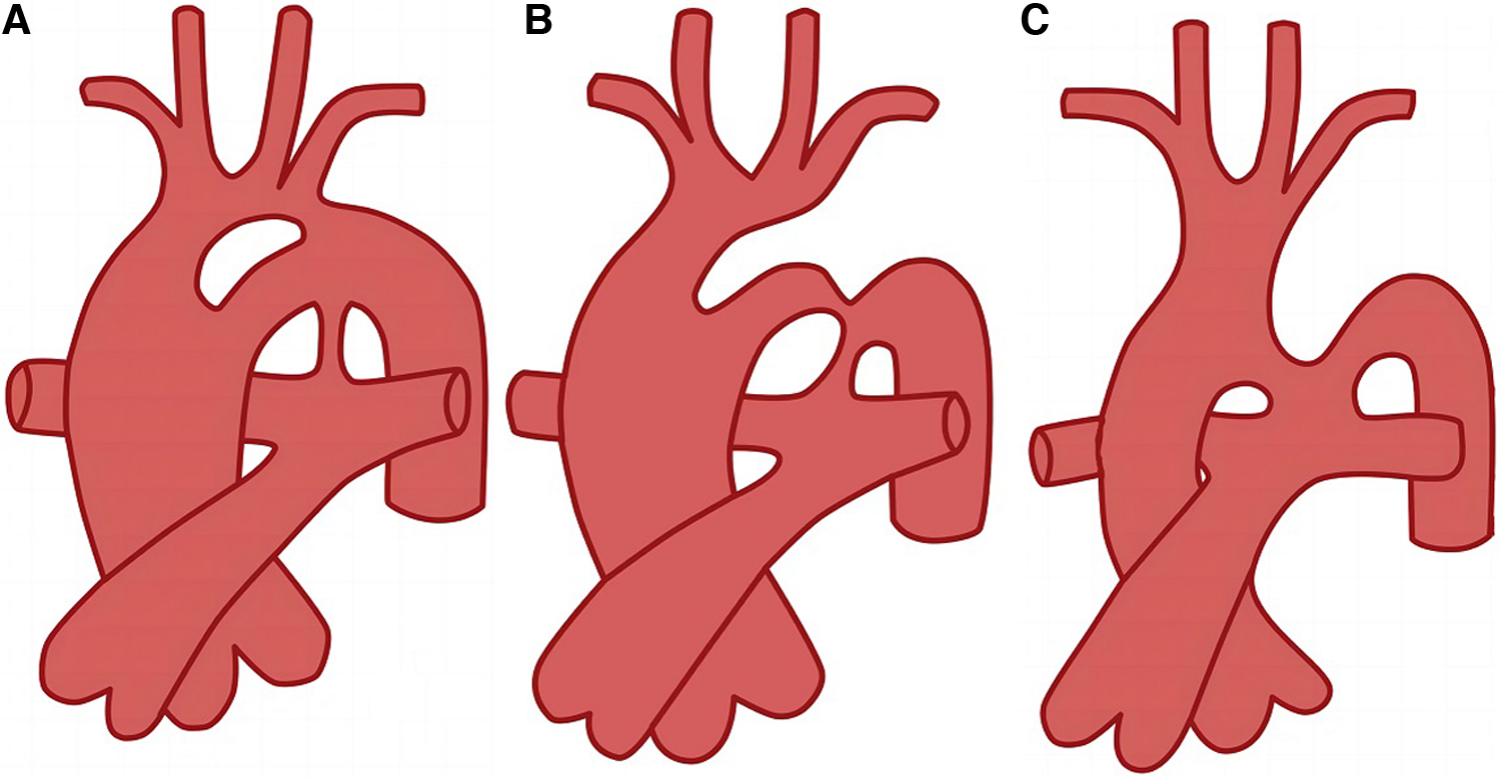
Diagram of Weinberg classification (5). (**A**) Weinberg type A, double-lumen aortic arch; (**B**) Weinberg type B, single-lumen aortic arch; (**C**) Weinberg type C, pulmonary to descending aorta.

As PFAA can occur unilaterally or bilaterally, it can present as various clinical types. The Weinberg classification is commonly used in clinical practice to categorise PFAA based on different abnormal vascular connections (5) (Figure 2). Weinberg type A is characterised by a double-lumen aortic arch, where the upper arch is the fourth arch, and the lower arch is the fifth arch, known as PFAA. The right innominate, left carotid, and left subclavian arteries typically arise from the fourth aortic arch. Weinberg type B is characterised by a single-lumen aortic arch, with the fourth aortic arch being interrupted, and the fifth arch originating from the ascending aorta and connecting to the descending aorta. Weinberg type C is characterised by the fifth arch originating from the proximal innominate artery of the ascending aorta and connecting to the pulmonary artery (Table 1).

**Table 1:** Comparison of improved classification and weinberg classification of PFAA.

Improved classification		Weinberg classification
A systemic to systemic connection		
A1 Double—lumen aortic arch (13, 14, 15–22)	**A**	Double lumen aortic arch
A2 Single-lumen aortic arch (8, 23–28)	**B**	The fifth arch remains, and the fourth arch is interruption
A3 Abnormal brachiocephalic artery origin (29)		
B systemic to pulmonary connection		
B1 With pulmonary obstruction (30–33)	**C**	The fifth arch originates from the proximal innominate artery of the ascending aorta and connects to the pulmonary via the sixth arch
B2 Without pulmonary obstruction (13, 30, 34–37)		
B3 With unrestricted systemic and pulmonary flows (9)		
C pulmonary to systemic connection		
With Aortic atresia (38)		
D hybrid		
Bilateral PFAA (39)		

PFAA, persistent fifth aortic arch; AAO, aorta ascendens.

In the present study, we conducted a literature search and collected data on 104 reported cases of PFAA from 77articles. We accurately classified these cases according to the Weinberg classification, excluding cases with unclear descriptions of the images and arterial connections. Among the 104 cases, Weinberg type A was the most common type of PFAA, accounting for 40.4% (42/104) of the cases, followed by Weinberg type B, accounting for 38.5% (40/104) of the cases. Weinberg type C was the least common, accounting for 18.3% (19/104) of the cases; the remaining 3 cases could not be classified using the Weinberg classification.

**Figure 3:**
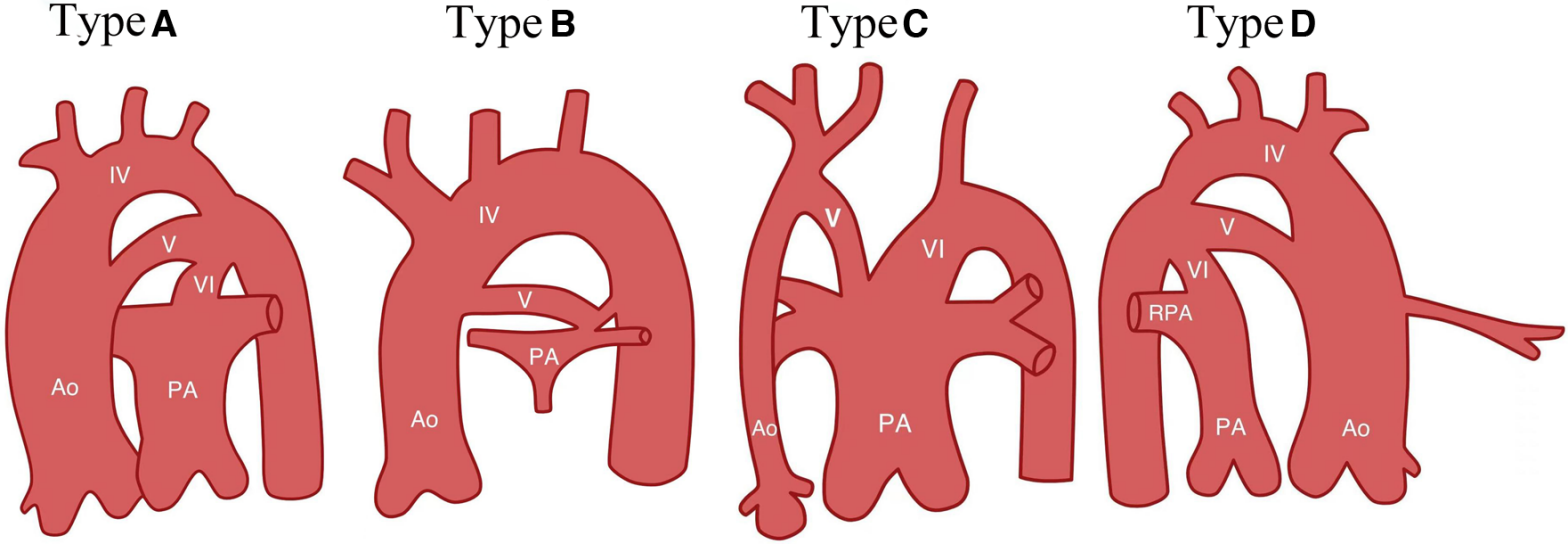
Diagram of improved classification (7). Type A, systemic-to-systemic connection; Type B, systemic-to-pulmonary connection; Type C, pulmonary-to-systemic connection; Type D, Bilateral PFAA, with right double-lumen aortic arch and left PFAA. Ao, ascending aortic arch; IV, aortic arch; V: fifth aortic arch; PA, pulmonary artery; RPA, right pulmonary artery; and VI, sixth branchial arch (arterial ducts).

Subsequently, as more cases of PFAA were reported and a deeper understanding of the condition was gained, many scholars began to suggest that the Weinberg classification could not sufficiently cover the various types of PFAA. Therefore, they proposed an improved classification based on the anatomical origin and haemodynamic changes of PFAA (6, 7, 40). This improved classification divides PFAA into four types (Figure 3 and Table 1).

Table 1 shows that the A1 type of the improved classification is equivalent to Weinberg type A, as both are characterised by a double-lumen aortic arch. The A2 type of the improved classification is equivalent to Weinberg type B, which is a single-lumen aortic arch, with the fourth arterial arch occluded or interrupted and the fifth aortic arch serving as the arterial arch supplying blood between the aorta and descending aorta. The B type of the improved classification is equivalent to Weinberg type C, where PFAA is connected from the ascending aorta and terminates in the pulmonary artery. Table 2 shows that the modified classification could cover all of our cases, including the 3 cases that were not defined by Weinberg classification, demonstrating that the relationship between the improved and Weinberg classifications is not a purely one-to-one correspondence. The improved classification covers the Weinberg classification and is relatively more comprehensive and specific. Therefore, the improved classification is a supplement and derivative based on the Weinberg classification, which can more completely summarise different common and rare types of PFAA. The Weinberg classification only covers the common PFAA types corresponding to the improved classification.

**Table 2:** Characteristics of the 104 cases of PFAA reported in the literature.

Age (case)	Sex (case)	Weinberg Classification (case)	Improved classification (case)	Diagnosis (case)	Treatment (case)
≤10 years (88)	Male (59)	Type A (42)	A1 (42)	US (18)	Surgical repair (52)
		Type B (40)	A2 (40)	CTA (39)	PGE1 + surgery (12)
	Female (39)	Type C (19)	A3 (1)	CAG (23)	Balloon Dilatation (4)
>10 years (12)			B1 (5)	MRA (5)	Stent implantation (5)
	Unclear (7)	Cannot define (3)	B2 (12)	Surgery (10)	No intervention (8)
			B3 (2)		PGE1 (2)
Unclear (4)			C(1)	Post-mortem (9)	Drug therapy (1)
			D(1)		Balloon + Stent (1)
					Unclear (19)

The numbers in parentheses represent the number of cases.

## 4. Clinical symptoms and complications

The clinical symptoms of PFAA are nonspecific and mainly depend on the combination of other deformities and their haemodynamic changes (41). Patients usually do not exhibit any obvious clinical symptoms when PFAA is not associated with other vascular malformations, and they may even be asymptomatic. This condition is more common in Weinberg type A patients, who are often diagnosed during physical or accidental examinations (12, 19, 42).

Patients with congenital heart disease, such as aortic atresia, interruption of aortic arch (IAA), coarctation of the aorta, patent ductus arteriosus (PDA), pulmonary atresia (PA), tricuspid atresia, atrial septal defect, and ventricular septal defect (VSD), may experience a range of symptoms, including heart murmur, cyanosis, cough, shortness of breath, pneumonia, and bronchitis. For instance, Li et al. (43) reported a 50-day-old boy with Weinberg type B PFAA who had IAA and PDA and presented with shortness of breath and abnormal heart murmur. Interestingly, for some patients with PFAA who are associated with PA, the presence of PFAA can be beneficial, as the fifth arch serves as an important system for pulmonary shunt (31, 33). However, severe cases may result in congestive heart failure, cardiogenic shock, and multiorgan failure (35, 44). For instance, Sinha et al. (33) reported the case of a 2-month-old boy with Weinberg type C PFAA who had a VSD and PA. The patient presented with central cyanosis, poor feeding, and failure to thrive.

Furthermore, some patients with PFAA may exhibit distinct facial and bodily abnormalities in clinical practice, which may be linked to genetic and chromosomal abnormalities, such as 22q11.2 chromosome deletion (16, 20, 45, 46), PHACE (25), and Cornelia de Lange syndromes (CdLS) (47). Based on our literature review, six cases of PFAA were associated with 22q11.2 chromosome deletion syndrome, and they primarily presented with scoliosis (48), palate abnormalities, facial deformities, hypocalcaemia (46), T-cell-mediated immune deficiency (49), and mild-to-moderate growth retardation. Growth retardation and poor feeding were the most common symptoms. In addition, two PFAA patients were found to have CdLS (14, 50) and were classified as Weinberg type A. CdLS is a genetic syndrome typically characterised by intellectual disability, distinct facial features, upper limb abnormalities, and atypical growth (51). In contrast, only one PFAA patient has been reported to have PHACE syndrome. The patient was classified as Weinberg type B and was associated with aneurysmal dilatation of the fourth aortic arch (25). The patient exhibited typical clinical manifestations of PHACE syndrome, such as frontotemporal cutaneous haemangioma, posterior fossa malformation, arterial abnormality, cardiac defects, and ocular abnormalities (52). Furthermore, the repeats of 22q11.2 (20) and 9q (47) are associated with PFAA. Therefore, clinicians should consider the possibility of PFAA in cases where a patient exhibits distinct facial features and abnormal cardiac haemodynamics.

## 5. Diagnostic imaging

Imaging technology plays a crucial role in the diagnosis of PFAA. Echocardiography is a non-invasive and cost-effective imaging method that allows real-time evaluation of dynamic anatomical structures, haemodynamics, and cardiac functions. It is recognised as the first-line diagnostic tool for PFAA, even in foetuses, owing to its ability to provide detailed information. Furthermore, echocardiography can be used to assess the systolic function of blood vessels preoperatively and postoperatively. However, complex extracardiac anatomy may lead to missed or incorrect diagnoses (8, 10). Computed tomography angiography (CTA) is considered to be more accurate than ultrasound in showing vascular origination, branching, aortic arch complexities, surrounding blood vessels, and their spatial structure. However, CTA is less effective in assessing intravascular blood flow. Magnetic resonance angiography (MRA) provides a clearer anatomical structure of the arch of aorta and its relationship with surrounding structures, thus making it a useful diagnostic tool before surgery or cardiac catheterisation. MRA has gradually replaced cardiac catheterisation as an effective non-invasive method in clinical practice. However, MRA application in clinical practice is limited by its high cost. Digital subtraction angiography is considered the gold standard for the diagnosis of PFAA (53); however, it involves radiation exposure and intervention (54).

Of the 104 cases we reviewed, almost all patients underwent echocardiography. According to our statistical analysis (Table 2), 18(17.3%) patients were initially diagnosed using echocardiography, 39(37.5%) patients were initially diagnosed using CTA, 23(22.1%) patients were initially diagnosed using cardiac catheterisation, 5(4.8%) patients were confirmed using MRA (29, 30, 38, 55, 56), 10(9.6%) patients were diagnosed during the operation, and 9(8.7%) patients were confirmed post-mortem (2–4, 9, 31, 57). The results indicate that various imaging techniques have great diagnostic values in identifying cardiovascular malformations. Echocardiography can serve as the first-line diagnostic tool for PFAA, and when combined with CTA and/or MRA, it can provide a more accurate evaluation of the pathological anatomy, clinical type, and haemodynamics of PFAA.

## 6. Treatment of PFAA

Patients with PFAA and no clinical symptoms may not require intervention. However, when PFAA is associated with other cardiovascular malformations caused severe haemodynamic change, surgical treatment and prostaglandin E1(PGE1) are often used to manage severe clinical symptoms in infancy (28). PGE1 is used to dilate blood vessels, reduce blood pressure, and prevent platelet aggregation (58). The surgical procedure typically involves resection of the narrow part or ligation of the fifth arch to reconstruct the fourth arch, patch enlargement of the arch stenosis, and patch augmentation of the fifth arch to replace the fourth arch, sometimes using the Gore-Tex tube grafts or stenting (8, 47, 59–61).

Of the 104 cases, 64 have undergone surgery and six patients underwent resection of the stenotic segment of the fifth arch and end-to-end anastomosis of the fifth arch with the descending aorta. In the present review, 14 patients received PGE1 treatment and 12 of them were subsequently treated surgically. Among the 14 patients who received PGE1 treatment (8, 38), except for the two patients who showed ineffective for PEG1 treatment, 12 patients showed good results in dilating the narrowed blood vessels (3, 15, 21, 23, 28, 30, 34, 58, 62–65). In our reviewed 104 cases,five patients underwent interventional balloon dilation; among them, two patients experienced long-term restenosis and required secondary balloon dilation or surgery (66, 67). Six patients received stent implants, and follow-up results showed normal ventricular ejection function and no definitive stenosis (24, 42, 47, 66, 68, 69).

## 7. Conclusion

PFAA is a rare congenital cardiovascular malformation typically reported as individual case studies. The clinical symptoms of PFAA are nonspecific and mainly depend on its complications. Due to the inadequate understanding of PFAA, patients with this condition are often misdiagnosed or missed diagnosed. In this review, we collected literature on reported cases of PFAA and summarised its embryonic development, pathological classification, imaging diagnosis, and clinical treatment. We aimed to provide a comprehensive understanding of PFAA and improve its diagnosis and treatment.

## References

[1] Edwin F. Aortic arch anomalies–persistent fifth aortic arch remnant. Interact Cardiovasc Thorac Surg. (2011) 12(1):71–2. 10.1510/icvts.2010.246462A 21177305

[2] Van Praagh R Van Praagh S. Persistent fifth arterial arch in man. Congenital double-lumen aortic arch. Am J Cardiol. (1969) 24(2):279–82. 10.1016/0002-9149(69)90417-2 5799089

[3] Gerlis LM Ho SY Anderson RH Da Costa P. Persistent 5th aortic arch–a great pretender: three new covert cases. Int J Cardiol. (1989) 23(2):239–47. 10.1016/0167-5273(89)90253-2 2656542

[4] Geva T Ray RA Santini F Van Praagh S Van Praagh R. Asymptomatic persistent fifth aortic arch (congenital double-lumen aortic arch) in an adult. Am J Cardiol. (1990) 65(20):1406–7. 10.1016/0002-9149(90)91339-8 2343831

[5] Weinberg PM. Aortic arch anomalies. J Cardiovasc Magn Reson. (2006) 8(4):633–43. 10.1080/10976640600713756 16869315

[6] Oppido G Davies B. Subclavian artery from ascending aorta or as the first branch of the aortic arch: another variant of persistent fifth aortic arch. J Thorac Cardiovasc Surg. (2006) 132(3):730–1. 10.1016/j.jtcvs.2006.03.065 16935159

[7] Freedom RM Yoo S-j Mikailian H Williams WG. The natural and modified history of congenital heart disease. Toronto, Canada: John Wiley & Sons (2008).

[8] Binsalamah ZM Chen P McKenzie ED. Aortic arch advancement for type a interrupted aortic arch with persistent fifth aortic arch type B. Cardiol Young. (2017) 27(5):1018–21. 10.1017/s1047951117000051 28260541

[9] Chiu CC Wu JR Chen HM Lin YT. Persistent fifth aortic arch: an ignored and underestimated disease. Jpn Heart J. (2000) 41(5):665–71. 10.1536/jhj.41.665 11132173

[10] Liu Y Zhang H Ren J Cao A Guo J Liu B Persistent fifth aortic arch: a single-center experience, case series. Transl Pediatr. (2021) 10(6):1566–72. 10.21037/tp-20-433 34295771 PMC8261588

[11] Marcus BS Rubio A Deen JF. Transcatheter relief of coarctation of the aorta in a persistent fifth aortic arch anatomy. Prog Pediatr Cardiol. (2020) 57:101200. 10.1016/j.ppedcard.2020.101200

[12] Rajagopal R Garg PK Khera PS Sharma S. “Double-Lumen” aortic arch with “double-lumen” brachiocephalic artery. Ann Pediatr Cardiol. (2019) 12(2):141–3. 10.4103/apc.APC_106_18 31143041 PMC6521648

[13] Yang H Zhu X Wu C Zhao X Ji X. Assessment of persistent fifth aortic arch by echocardiography and computed tomography angiography. Medicine (Baltimore). (2020) 99(9):e19297. 10.1097/md.0000000000019297 32118745 PMC7478403

[14] Naimo PS Vazquez-Alvarez Mdel C d'Udekem Y Jones B Konstantinov IE. Double-Lumen aortic arch: persistence of the fifth aortic arch. Ann Thorac Surg. (2016) 101(5):e155–6. 10.1016/j.athoracsur.2015.10.014 27106464

[15] Lambert V Blaysat G Sidi D Lacour-Gayet F. Double-Lumen aortic arch by persistence of fifth aortic arch: a new case associated with coarctation. Pediatr Cardiol. (1999) 20(2):167–9. 10.1007/s002469900431 9986901

[16] Oshitani T Kawasaki Y Murakami Y Fujino M Sasaki T Nakamura K A double-barrelled aorta with high aortic arch. J Cardiol Cases. (2021) 24(6):284–6. 10.1016/j.jccase.2021.05.003 34917211 PMC8642631

[17] Linhares RR Silva CE Monaco CG Ferreira LD Gil MA Ortiz J Double lumen aortic arch or persistence of fifth aortic arch?- report of a case with No associated cardiac defects and literature review. Echocardiography (Mount Kisco, NY). (2011) 28(7):E143–5. 10.1111/j.1540-8175.2011.01415.x 21843253

[18] Bernasconi A Goo HW Yoo SJ. Double-Barrelled aorta with tetralogy of fallot and pulmonary atresia. Cardiol Young. (2007) 17(1):98–101. 10.1017/s104795110600148x 17184561

[19] Tehrai M Saidi B Goudarzi M. Multi-Detector computed tomography demonstration of double-lumen aortic arch–persistent fifth arch–as an isolated anomaly in an adult. Cardiol Young. (2012) 22(3):353–5. 10.1017/s1047951111001855 22067220

[20] Boggs RA Romp RL. Persistent fifth aortic arch confirmed by computed tomography angiography. World J Pediatr Congenit Heart Surg. (2015) 6(4):670–1. 10.1177/2150135115593135 26467886

[21] Nishi K Inamura N Marutani S Nishino T Takemura T. Rare basis of patent ductus arteriosus: persistence of the fifth aortic arch. Pediatr Int. (2017) 59(10):1091–3. 10.1111/ped.13379 29081079

[22] Tretter JT Crotty EJ Anderson RH Taylor MD. How should we diagnose persistence of the artery of the fifth pharyngeal arch? Pediatr Cardiol. (2017) 38(8):1722–4. 10.1007/s00246-017-1665-y 28639150

[23] Cetrano E Polito A Trezzi M Carotti A. Neonatal repair of persistent fifth aortic arch coarctation and interrupted fourth aortic arch. Ann Thorac Surg. (2017) 103(5):e475–e7. 10.1016/j.athoracsur.2016.11.085 28431733

[24] Kato A Ohashi N Nishikawa H. A case report: stent implantation to treat coarctation of persistent 5th aortic arch associated with interrupted 4th aortic arch. Eur Heart J Case Rep. (2019) 3(2):ytz076. 10.1093/ehjcr/ytz076 31449629 PMC6601166

[25] Chiappa E Greco A Fainardi V Passantino S Serranti D Favilli S. Aortic arch interruption and persistent fifth aortic arch in phace syndrome: prenatal diagnosis and postnatal course. Echocardiography (Mount Kisco, NY). (2015) 32(9):1441–3. 10.1111/echo.12941 25809619

[26] Schicchi N Agliata G Giovagnoni A. Ct imaging of a rare case of persistent fifth aortic arch in newborn. BJR case Rep. (2016) 2(2):20150048. 10.1259/bjrcr.20150048 30363644 PMC6180890

[27] Wei X Wang F Cao R An Q. True bovine aortic arch combined with coarctation of persistent fifth aortic arch in a child. Eur Heart J Cardiovasc Imaging. (2019) 20(7):837. 10.1093/ehjci/jez006 30726892

[28] Zartner P Schneider MB Bein G. Prostaglandin E1 sensitive persistent fifth aortic arch type 2. Heart (British Cardiac Society). (2000) 84(2):142. 10.1136/heart.84.2.141 10979721 PMC1760913

[29] McMahon CJ Kertesz NJ Vick GW. Delineation of persistent fifth aortic arch using magnetic resonance angiography. Cardiol Young. (2002) 12(5):484–5. 10.1017/s1047951102000835 15773454

[30] Khan S Nihill MR. Clinical presentation of persistent 5th aortic arch: 3 new cases. Tex Heart Inst J. (2006) 33(3):361–4. 17041697 PMC1592264

[31] Gerlis LM Dickinson DF Wilson N Gibbs JL. Persistent fifth aortic arch. A report of two new cases and a review of the literature. Int J Cardiol. (1987) 16(2):185–92. 10.1016/0167-5273(87)90250-6 3623724

[32] Subramanyan R Sahayaraj A Sekar P Cherian KM. Persistent fifth aortic arch. J Thorac Cardiovasc Surg. (2010) 139(6):e117–8. 10.1016/j.jtcvs.2009.04.028 19660414

[33] Sinha M Rajagopal R Pandey NN Kumar S. Type 2 persistent fifth aortic arch: an elusive entity diagnosed on computed tomography angiography. J Cardiovasc Comput Tomogr. (2020) 14(5):e29–30. 10.1016/j.jcct.2018.11.002 30415961

[34] Gupta SK Gulati GS Anderson RH. Clarifying the anatomy of the fifth arch artery. Ann Pediatr Cardiol. (2016) 9(1):62–7. 10.4103/0974-2069.171392 27011696 PMC4782472

[35] Meliota G Lombardi M Zaza P Tagliente MR Versacci P Scalzo G Isolated persistence of the fifth aortic arch in an infant presenting with congestive heart failure. Ann Pediatr Cardiol. (2020) 13(1):91–4. 10.4103/apc.APC_53_19 32030044 PMC6979032

[36] Hwang MS Chang YS Chu JJ Su WJ. Isolated persistent fifth aortic arch with systemic-to-pulmonary arterial connection. J Thorac Cardiovasc Surg. (2003) 126(5):1643–4. 10.1016/s0022-5223(03)00954-1 14666049

[37] Murugan MK Gulati GS Saxena A Juneja R Gupta SK. Multi-detector computed tomography (Mdct) in persistent fifth aortic arch (Pfaa). Heart Lung Circ. (2014) 23(2):e71–3. 10.1016/j.hlc.2013.05.649 23932505

[38] Bhatla P Chakravarti S Axel L Ludomirsky A Revah G. Prenatal diagnosis of a persistent fifth aortic arch, pulmonary-to-systemic type: an unusual association with evolving aortic coarctation. Echocardiography (Mount Kisco, NY). (2015) 32(5):875–7. 10.1111/echo.12850 25418608

[39] Wang JN Wu JM Yang YJ. Double-Lumen aortic arch with anomalous left pulmonary artery origin from the main pulmonary artery–bilateral persistent fifth aortic arch–a case report. Int J Cardiol. (1999) 69(1):105–8. 10.1016/s0167-5273(99)00011-x 10362382

[40] Moes CA Freedom RM. Rare types of aortic arch anomalies. Pediatr Cardiol. (1993) 14(2):93–101. 10.1007/bf00796987 8469639

[41] Valderrama P Álvarez T Ballesteros F Rodríguez A Zunzunegui JL. Coarctation of persistent fifth aortic arch with interrupted fourth arch: first pediatric report of stent intervention. Rev Esp Cardiol (English ed). (2016) 69(3):337–8. 10.1016/j.rec.2015.10.021 26778595

[42] Gangadhara M. Incidental detection of persistent fifth aortic arch in a 19-year-old gentleman with coarctation of the aorta. Cardiol. (2019) 1:104.

[43] Li X Li J Qin W Zhang R. Surgical repair of persistent fifth aortic arch coarctation and interrupted fourth aortic arch without cardiopulmonary bypass: a case report. Transl Pediatr. (2022) 11(2):306–10. 10.21037/tp-21-350 35282028 PMC8905098

[44] Jurí R Alday LE De Rossi R. Interrupted fourth aortic arch with persistent fifth aortic arch and aortic coarctation—treatment with balloon angioplasty combined with surgery. Cardiol Young. (1994) 4(3):304–6. 10.1017/S1047951100011239

[45] Bernheimer J Friedberg M Chan F Silverman N. Echocardiographic diagnosis of persistent fifth aortic arch. Echocardiography (Mount Kisco, NY). (2007) 24(3):258–62. 10.1111/j.1540-8175.2007.00383.x 17313637

[46] Lee ML Chen HN Chen M Tsao LY Wang BT Lee MH Persistent fifth aortic arch associated with 22q11.2 deletion syndrome. J Formos Med Assoc. (2006) 105(4):284–9. 10.1016/s0929-6646(09)60119-4 16618608

[47] Figueras-Coll M Sabaté-Rotés A Betrián-Blasco P Ortuño-Muro P. Stenting coarctation of the “fifth aortic arch": a safe and attractive therapeutic alternative to surgery. World J Pediatr Congenit Heart Surg. (2020) 11(4):Np140-np3. 10.1177/2150135117752895 29614911

[48] de Reuver S Homans JF Schlösser TPC Houben ML Deeney VFX Crowley TB 22q11.2 Deletion syndrome as a human model for idiopathic scoliosis. J Clin Med. (2021) 10(21):4823. 10.3390/jcm10214823 34768342 PMC8584329

[49] Zhang Z Shi L Song L Maurer K Zhao X Zackai EH Chromatin modifications in 22q11.2 deletion syndrome. J Clin Immunol. (2021) 41(8):1853–64. 10.1007/s10875-021-01123-2 34435264

[50] Park HJ Oh JM Park SE Nam SO Kim CW Kim KI. Isolated persistent fifth aortic arch in a patient with cornelia De lange syndrome. Pediatr Cardiol. (2005) 26(1):112–4. 10.1007/s00246-004-9012-5 15565268

[51] Kline AD Moss JF Selicorni A Bisgaard AM Deardorff MA Gillett PM Diagnosis and management of cornelia De lange syndrome: first international consensus statement. Nat Rev Genet. (2018) 19(10):649–66. 10.1038/s41576-018-0031-0 29995837 PMC7136165

[52] Chamli A Litaiem N. Phace syndrome. Statpearls. Treasure Island, FL: StatPearls Publishing Copyright © 2022, StatPearls Publishing LLC (2022).

[53] Camarda JA Russell DS Frommelt M. Persistent fifth aortic arch: echocardiographic diagnosis of a persistent fifth aortic arch. Echocardiography (Mount Kisco, NY). (2011) 28(2):E44–5. 10.1111/j.1540-8175.2010.01324.x 21083758

[54] Zheng L Cao YL Wu RC Guo J Ma N Wang FY Persistent fifth aortic arch stenosis associated with type a interruption of the aortic arch: a report of six cases. Chin Med J. (2019) 132(12):1482–4. 10.1097/cm9.0000000000000278 31205108 PMC6629331

[55] Kirsch J Julsrud PR. Magnetic resonance angiography of an ipsilateral double aortic arch due to persistent left fourth and fifth aortic arches. Pediatr Radiol. (2007) 37(5):501–2. 10.1007/s00247-007-0437-x 17415604

[56] Yang SG Fogel MA Stephens P Jr. Bellah RD Weinberg PM. Noninvasive imaging of isolated persistent fifth aortic arch. Pediatr Cardiol. (2003) 24(2):179–81. 10.1007/s00246-002-2361-z 12574973

[57] Parmar RC Pillai S Kulkarni S Sivaraman A. Type I persistent left fifth aortic arch with truncus arteriosus type A3: an unreported association. Pediatr Cardiol. (2004) 25(4):432–3. 10.1007/s00246-003-9008-6 15054549

[58] Carroll SJ Ferris A Chen J Liberman L. Efficacy of prostaglandin E1 in relieving obstruction in coarctation of a persistent fifth aortic arch without opening the ductus arteriosus. Pediatr Cardiol. (2006) 27(6):766–8. 10.1007/s00246-006-1380-6 17111291

[59] Kim SH Choi ES Cho S Kim WH. Persistent fifth aortic arch with coarctation. Korean J Thorac Cardiovasc Surg. (2016) 49(1):39–41. 10.5090/kjtcs.2016.49.1.39 26889445 PMC4757396

[60] Tang X Wang L Wu Q Tong X. Persistent fifth aortic arch with interrupted aortic arch. J Card Surg. (2015) 30(3):284–7. 10.1111/jocs.12450 25195807

[61] Herrera MA D’Souza VJ Link KM Weesner KM Formanek AG. A persistent fifth aortic arch in man: a double-lumen aortic arch (presentation of a new case and review of the literature). Pediatr Cardiol. (1987) 8(4):265–9. 10.1007/bf02427540 3324071

[62] Nakashima K Oka N Hayashi H Shibata M Kitamura T Itatani K A case report of persistent fifth aortic arch presenting with severe left ventricular dysfunction. Int Heart J. (2014) 55(1):87–8. 10.1536/ihj.13-167 24463916

[63] Santoro G Caianiello G Palladino MT Iacono C Russo MG Calabrò R. Aortic coarctation with persistent fifth left aortic arch. Int J Cardiol. (2009) 136(2):e33–4. 10.1016/j.ijcard.2008.04.091 18692918

[64] Iwase J Maeda M Sasaki S Mizuno A. Images in cardiothoracic surgery. Persistent fifth aortic arch. Ann Thorac Surg. (2006) 81(5):1908. 10.1016/j.athoracsur.2004.07.068 16631709

[65] Divekar AA Sebastian VA. Neonatal repair of persistent fifth aortic arch and aortic coarctation. JTCVS Tech. (2020) 4:245–6. 10.1016/j.xjtc.2020.09.022 34318029 PMC8307882

[66] Uysal F Bostan OM Cil E. Coarctation of persistent 5th aortic arch: first report of catheter-based intervention. Tex Heart Inst J. (2014) 41(4):411–3. 10.14503/thij-13-3385 25120395 PMC4120505

[67] Da Costa AG Iwahashi ER Atik E Rati MA Ebaid M. Persistence of hypoplastic and recoarcted fifth aortic arch associated with type a aortic arch interruption: surgical and balloon angioplasty results in an infant. Pediatr Cardiol. (1992) 13(2):104–6. 10.1007/bf00798215 1535439

[68] Koch A Ludwig J Zink S Singer H. Isolated left pulmonary artery: interventional stenting of a persistent fifth aortic arch. Catheter Cardiovasc Interv. (2007) 70(1):105–9. 10.1002/ccd.21099 17377997

[69] Khajali Z Ali Basiri H Maleki M. Persistent fifth aortic arch associated with coarctation of aorta: a case report. Congenit Heart Dis. (2011) 6(6):650–2. 10.1111/j.1747-0803.2011.00492.x 21435186

